# Epigenetic mechanisms mediate the experimental evolution of resistance against parasitic fungi in the greater wax moth *Galleria mellonella*

**DOI:** 10.1038/s41598-018-36829-8

**Published:** 2019-02-07

**Authors:** Krishnendu Mukherjee, Ivan Dubovskiy, Ekaterina Grizanova, Rüdiger Lehmann, Andreas Vilcinskas

**Affiliations:** 1 Fraunhofer Institute for Molecular Biology and Applied Ecology, Department of Bioresources, Winchester Str. 2, 35394 Giessen, Germany; 2 Novosibirsk State Agrarian University, Dobrolubova 160, 630039 Novosibirsk, Russia; 3 Institute for Insect Biotechnology, Justus-Liebig University of Giessen, Heinrich-Buff-Ring 26-32, 35392 Giessen, Germany

## Abstract

Recent concepts in evolutionary biology suggest that epigenetic mechanisms can translate environmental selection pressures into heritable changes in phenotype. To determine whether experimental selection for a complex trait in insects involves epigenetic modifications, we carried out a generation-spanning experiment using larvae of the greater wax moth *Galleria mellonella* as a model host to investigate the role of epigenetics in the heritability of resistance against the parasitic fungus *Metarhizium robertsii*. We investigated differences in DNA methylation, histone acetylation and microRNA (miRNA) expression between an experimentally resistant population and an unselected, susceptible line, revealing that the survival of *G*. *mellonella* larvae infected with *M*. *robertsii* correlates with tissue-specific changes in DNA methylation and histone modification and the modulation of genes encoding the corresponding enzymes. We also identified miRNAs differentially expressed between resistant and susceptible larvae and showed that these regulatory molecules target genes encoding proteinases and proteinase inhibitors, as well as genes related to cuticle composition, innate immunity and metabolism. These results support our hypothesis that epigenetic mechanisms facilitate, at least in part, the heritable manifestation of parasite resistance in insects. The reciprocal adaptations underlying host–parasite coevolution therefore extend beyond the genetic level to encompass epigenetic modifications.

## Introduction

Classical natural selection operates by favoring the survival and reproduction of the fittest phenotypes, which pass their adaptations to subsequent generations via genetic changes, i.e. mutations. However, evolutionary theory has more recently accommodated the possibility that heritable adaptations to environmental conditions can also be conferred by epigenetic mechanisms, which do not require changes in the DNA sequence^1–3^. Such mechanisms control the ability of transcription factors to access the genome, thereby facilitating rapid adaptations to changing environmental conditions, but this process is difficult to study in wild populations where selection pressure cannot be controlled^4,5^. The involvement of epigenetic mechanisms in evolutionary adaptations can be tested in the laboratory, e.g. by imposing artificial selection pressure over multiple generations in a suitable model system. The greater wax moth *Galleria mellonella* and its fungal parasites have recently been established as a model system for such generation-spanning experimental studies^6–8^.

Parasitic fungi such as *Metarhizium robertsii* (formerly *M*. *anisopliae*) differ from other entomopathogens by infecting insect hosts directly through the integument, a typically impenetrable physical barrier against microbial invaders^8,9^. This virulence-related trait is mediated by secreted proteolytic enzymes that not only penetrate the cuticle but also convert the insect host into a source of nutrients^10,11^. In turn, *G*. *mellonella* has evolved the ability to sense virulence-associated fungal enzymes and respond by synthesizing antifungal peptides such as gallerimycin^12^ and inhibitors of fungal proteinases^13–16^, thus synergistically combating the fungal infection^17^. *M*. *robertsii* has evolved the ability to sense such antifungal molecules and respond by synthesizing metalloproteinases as a means to degrade them^18^. As a further counter-adaptation, *G*. *mellonella* has evolved the capacity to sense the virulence-associated metalloproteinases and synthesize a specific inhibitor^19,20^. These reciprocal responses and counter-responses reveal a co-evolved communication network between the fungal parasite and its insect host as a means to decide the outcome of infection^18^. Such an arms race between fungal virulence factors and host immunity-related effector molecules during host–parasite coevolution is an ideal setting in which to study epigenetic mechanisms controlling the induction of fungal proteinases and host-derived proteinase inhibitors^7^.

The epigenetic regulation of gene expression involves the chemical modification of DNA and/or histones. The methylation of cytidine residues in DNA by DNA methyltransferases (DNMTs) produces 5-methylcytidine, which is associated with condensed, inaccessible chromatin and gene silencing^21^. Similarly, histone acetylation/deacetylation influences chromatin structure, reflecting the opposing activities of histone acetyltransferases (HATs) and histone deacetylases (HDACs). HATs open the chromatin structure, promoting access to the DNA and thus gene expression, whereas the condensed chromatin generated by HDACs causes gene silencing. Histone acetylation/deacetylation regulates transcription during metamorphosis, wounding and fungal infection in *G*. *mellonella*^22^. Gene expression in eukaryotes can also be regulated at the post-transcriptional level by short non-coding RNAs (∼18–24 nucleotides in length) known as microRNAs (miRNAs), which inhibit protein synthesis by base-pairing with the 3′ and 5′ untranslated regions (UTRs) of target messenger RNAs (mRNAs)^23–25^. The seed sequence or seed region (typically 2–8 nucleotides from the 5´-end of the miRNA) is essential for mRNA binding. We have recently confirmed that miRNAs in *G*. *mellonella* are also involved in the regulation of immunity^26^.

Here we tested the hypothesis that epigenetic mechanisms mediate the rapid adaptation of insects to overcome the threat of pathogens. We conducted generation-spanning experiments in *G*. *mellonella* larvae in which lines experimentally selected for resistance against *M*. *robertsii* were compared to unselected (susceptible) lines to determine the relative levels of DNA methylation, histone modification and miRNA expression. Our results highlighted the involvement of all three mechanisms in the establishment of heritable resistance.

## Results

### Resistance to *M*. *robertsii* is associated with tissue-specific differences in DNA methylation

**Figure 1:**
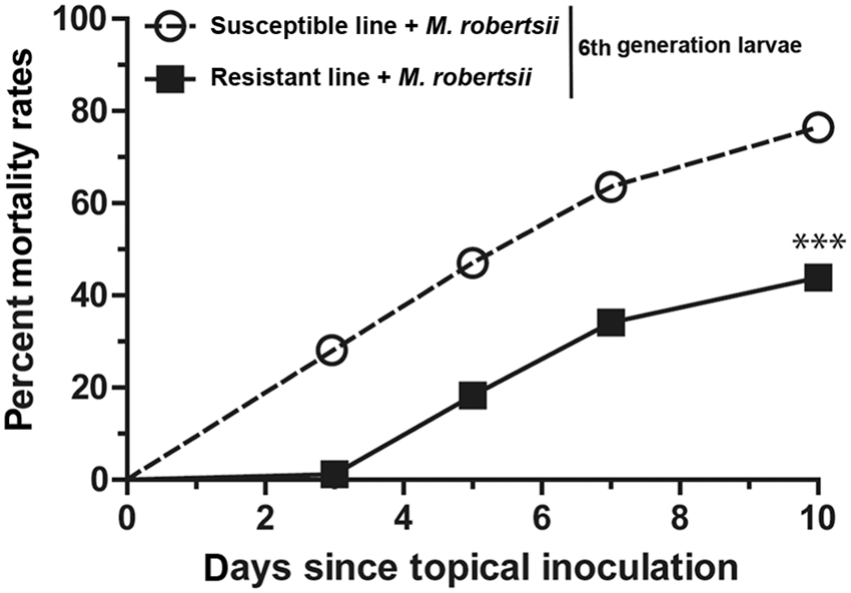
Mortality of *G*. *mellonella* larvae after inoculation with *M. robertsii* conidia. The mortality of resistant and susceptible larvae following inoculation with *M*. *robertsii* conidia (***p < 0.001).

**Figure 2:**
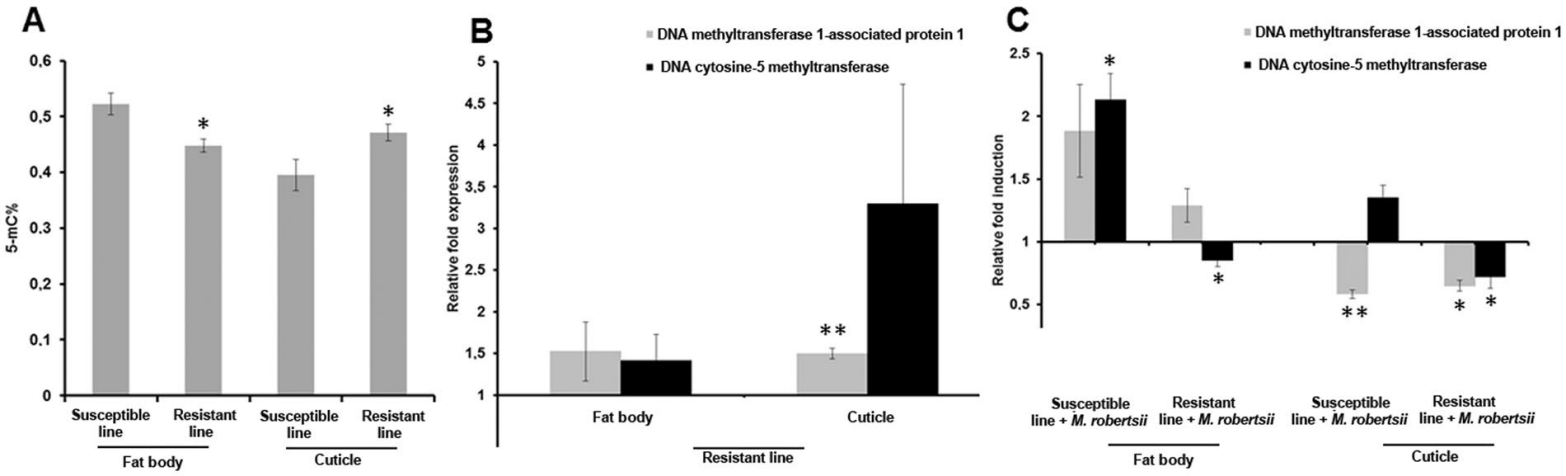
Tissue-specific differences in DNA methylation between resistant and susceptible *G*. *mellonella larvae*. (**A**) Global DNA methylation level in the fat body and cuticle of R^−^ and S^−^ larvae (*p < 0.05). (**B**,**C**) Expression level of the genes for DNA methyltransferase 1-associated protein 1 and DNA cytosine-5 methyltransferase in the fat body and cuticle of (**B**) R^−^ larvae relative to S^−^ larvae (**p < 0.005) and (**C**) R^+^/S^+^ larvae relative to R^−^/S^−^ counterparts (**p < 0.005, *p < 0.05). The 18 *S* rRNA housekeeping gene was used for internal data normalization. Data are means of three independent experiments with standard errors. Names of larval cohorts: R^+^ infected resistant; R^−^ uninfected resistant; S^+^ infected susceptible; S^−^ uninfected susceptible.

To determine whether the evolution of resistance against the parasitic fungus *M*. *robertsii* in the insect host *G*. *mellonella* is associated with changes in DNA methylation, we experimentally selected larvae for resistance over multiple generations and compared the resistant line with an unselected susceptible line. We infected *G*. *mellonella* larvae by inoculation with *M*. *robertsii* conidia, and survivors were allowed to breed over six generations, under the same selection pressure. In the sixth generation, the larvae from the selected line showed a 33% greater survival rate compared to larvae from the line propagated without selection (Fig. 1). The insect cuticle is the primary physiological barrier against penetrating hyphae^9^, and the fat body is required for a systemic immune response. To determine whether fungal resistance was associated with tissue-specific epigenetic reprogramming of innate immunity-related gene expression, we isolated DNA from the cuticle and fat body of the resistant and susceptible larvae for 5-methylcytosine quantification. The levels of 5-methylcytosine were higher in the cuticle but lower in the fat body of the resistant larvae compared to the susceptible larvae (Fig. 2A). Furthermore, global DNA methylation levels in susceptible larvae were higher in the fat body than the cuticle, but in the resistant larvae there was no significant difference between these tissues.

Next we investigated the effect of infecting the larvae with *M*. *robertsii* conidia, resulting in four cohorts for comparative analysis: uninfected resistant (R^−^), uninfected susceptible (S^−^), infected resistant (R^+^) and infected susceptible (S^+^). The DNA methyltransferase 1-associated protein 1 gene was upregulated in the cuticle of R^−^ compared to S^−^ larvae but the DNA cytosine 5-methyltransferase gene was not, and neither gene was significantly modulated in the fat body of R^−^ or S^−^ larvae (Fig. 2B). However, both genes were induced in the fat body of S^+^ larvae compared to S^−^ controls (Fig. 2C). In contrast, these genes were downregulated in the fat body and cuticle of R^+^ larvae compared to R^−^ controls (Fig. 2C).

### Resistance to *M*. *robertsii* is associated with tissue-specific differences in histone acetylation

**Figure 3:**
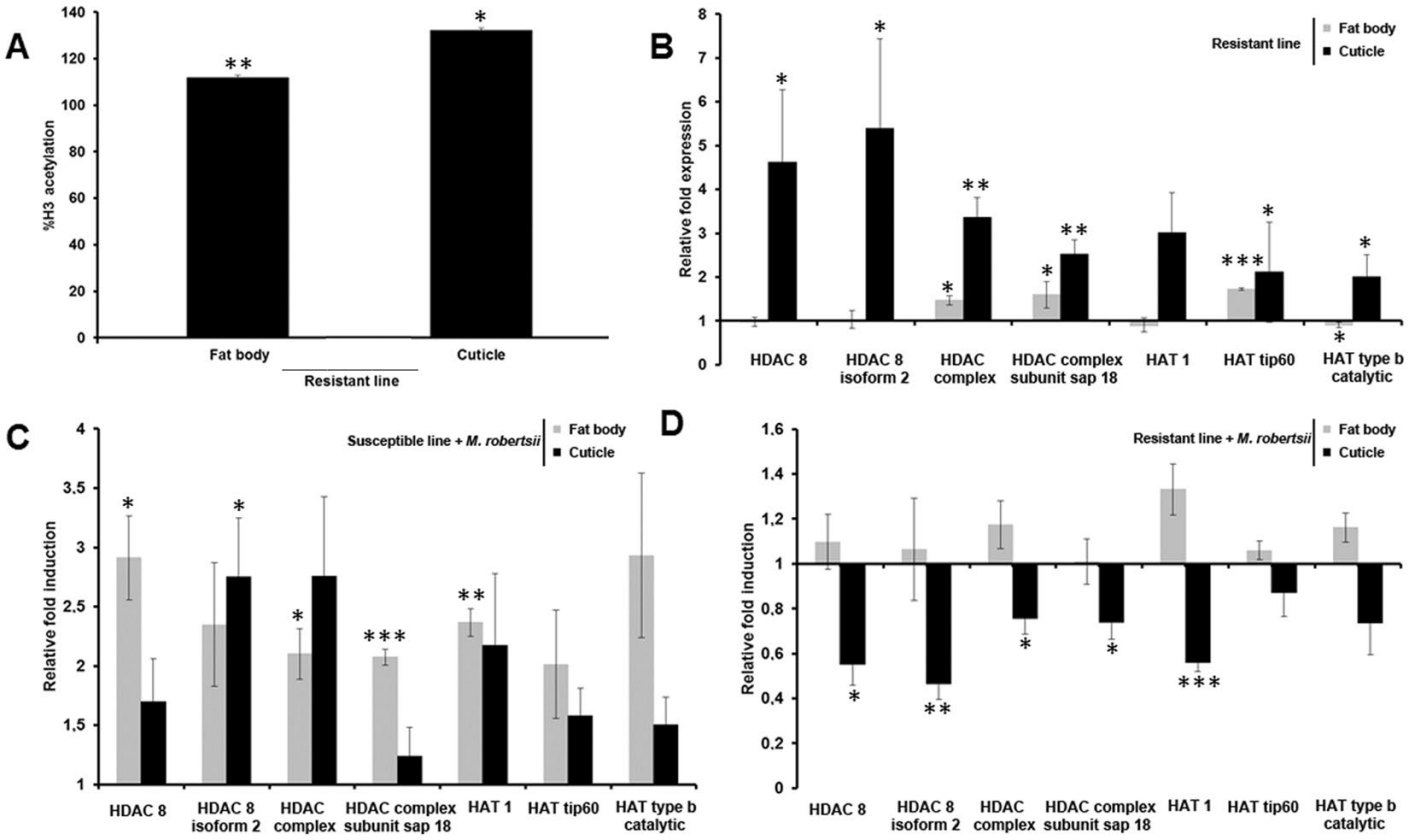
Tissue-specific differences in histone acetylation between resistant and susceptible *G*. *mellonella* larvae. (**A**) Global histone H3 acetylation levels in the cuticle and fat body of R^−^relative to S^−^ larvae. Data are means of three independent experiments with standard deviations (*p < 0.05, **p < 0.005). (**B–D**) Expression level of genes encoding HDAC 8, HDAC 8 isoform 2, HDAC complex, HDAC complex subunit sap18, HAT1, HAT tip60 and HAT type b catalytic in the fat body and cuticle of (**B**) R^−^ larvae relative to S^−^ larvae (*p < 0.005, **p < 0.005, ***p < 0.0005), (C) S^+^ larvae relative to S^−^ larvae (*p < 0.005, **p < 0.005, ***p < 0.0005), and (**D**) R^+^ larvae relative to R^−^ larvae(*p < 0.05, **p < 0.005, ***p < 0.0005). The 18 *S* rRNA housekeeping gene was used for internal data normalization. Data are means of three independent experiments with standard errors. Names of larval cohorts: R^+^ infected resistant; R^−^ uninfected resistant; S^+^ infected susceptible; S^−^ uninfected susceptible.

To determine whether the evolution of fungal resistance in *G*. *mellonella* also involves histone modification, we compared the acetylation of core histone H3 and the expression of genes encoding HDAC and HATs in the selected resistant line and unselected susceptible control line. The acetylation of core histone H3 was analyzed because this modification is involved in the transcriptional control of immunity and development in eukaryotes, and regulates the expression of immunity-related genes in insects that have evolved resistance to *Bacillus thuringiensis*^7^. We detected higher levels of histone H3 acetylation in the cuticle and fat body of the resistant larvae compared to the same tissues in the susceptible larvae (Fig. 3A). However, we did not detect differences in H3 acetylation between the cuticle and fat body in the resistant larvae (Fig. 3A).

Next we investigated the enzymatic modulators of histone acetylation in resistant larvae by measuring the expression of HDAC and HAT genes such as those encoding HDAC8, HDAC8 isoform 2, HDAC complex, HDAC complex subunit sap18, HAT1, HAT tip60, and HAT type b catalytic. The genes encoding HDAC complex, subunit sap18 and HAT tip60 were upregulated in the fat body and cuticle of R^−^ larvae compared to S^−^ larvae, whereas the gene encoding HAT type b catalytic was downregulated in the fat body of the R^−^ larvae (Fig. 3B). All three genes were upregulated in the cuticle and fat body of the S^+^ larvae but downregulated in the cuticle of the R^+^ larvae (Fig. 3C,D).

### Differential regulation of miRNAs in resistant and susceptible *G*. *mellonella* larvae

Previous studies have shown that *G*. *mellonella* miRNAs contribute to the post-transcriptional regulation of immune responses against parasitic fungi^26^. To determine whether miRNAs also mediate the evolution of resistance against *M*. *robertsii*, we compared the expression profiles of miRNAs in the selected resistant line and unselected susceptible control line using a DNA oligonucleotide microarray (Table S1) given that the *G*. *mellonella* genome sequence is not yet fully annotated^26,27^. All probes representing unique mature miRNAs were printed in triplicate for verification (Fig. S1).

We carried out all possible pairwise comparisons among the four cohorts to find miRNAs that were differentially expressed (Table S2). In the fat body, each pairwise comparison yielded more than 200 differentially expressed miRNAs (R^−^ *vs* S^−^ = 235, R^−^ *vs* S^+^  = 230; R^+^ *vs* S^−^ = 260, R^+^ *vs* S^+^  = 222, S^+^ *vs* S^−^ = 202). In the cuticle, there were more differentially expressed miRNAs than in the fat body for most comparisons (R^−^ *vs* S^−^ = 336, R^−^ *vs* S^+^  = 214; R^+^ *vs* S^−^ = 243, R^+^ *vs* S^+^  = 228, S^+^ *vs* S^−^ = 305, R^+^ *vs* R^−^ = 222). We also compared the miRNA expression profiles between the two tissues for each cohort. The number of miRNAs differentially expressed between the fat body and cuticle was 290 for the S^+^ cohort, 264 for the R^+^ cohort, 416 for the S^−^ cohort and 312 for the R^−^ cohort. Furthermore, 304 miRNAs were differentially expressed between the fat body and cuticle of the resistant and susceptible lines regardless of the infection state (Table S2).

**Figure 4:**
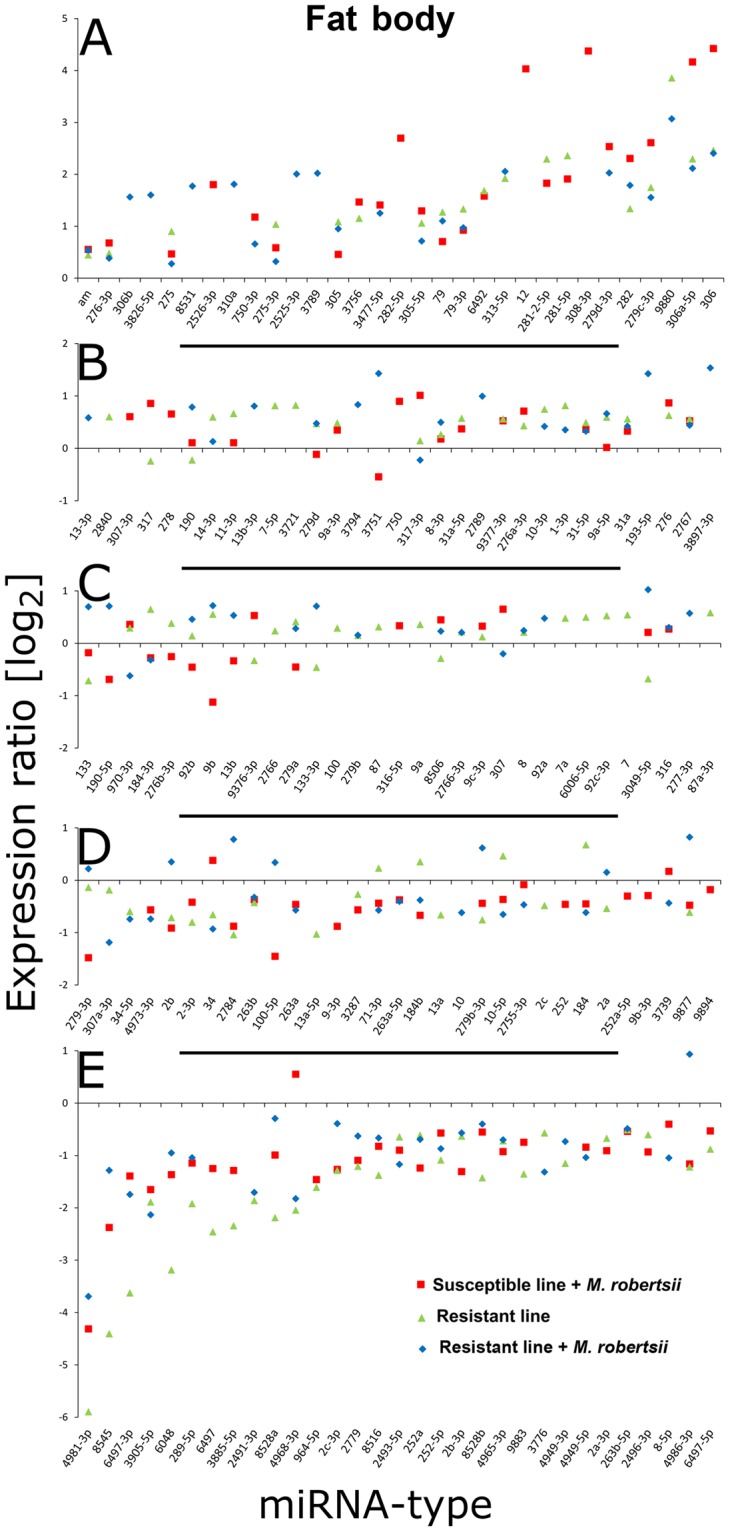
Distribution of miRNAs in the fat body of resistant and susceptible *G*. *mellonella* larvae infected with *M*. *robertsii*. The miRNA sequences were obtained from miRBase v21 and their expression profiles were determined by microarray analysis. (**A**–**E**) Identification of miRNAs that are differentially expressed in R^+^, R^−^ and S^+^ larvae, with fold changes in expression relative to S^−^ larvae. Names of larval cohorts: R^+^ infected resistant; R^−^ uninfected resistant; S^+^ infected susceptible; S^−^ uninfected susceptible.

**Figure 5:**
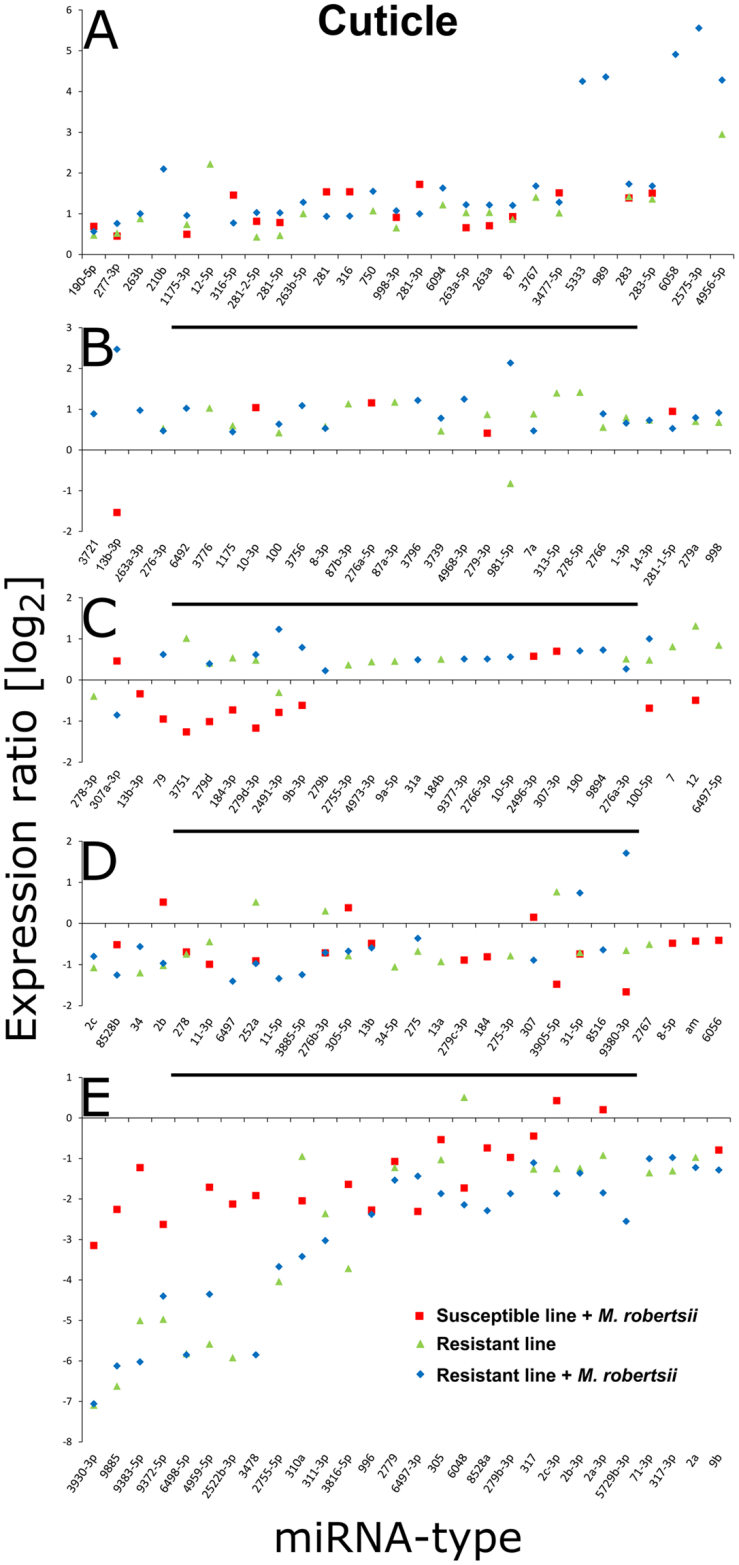
Distribution of miRNAs in the cuticle of resistant and susceptible *G*. *mellonella* larvae infected with *M*. *robertsii*. The miRNA sequences were obtained from miRBase v21 and their expression profiles were determined by microarray analysis. (**A**–**E**) Identification of miRNAs that are differentially expressed in R^+^, R^−^ and S^+^ larvae, with fold changes in expression relative to S^−^ larvae. Names of larval cohorts: R^+^ infected resistant; R^−^ uninfected resistant; S^+^ infected susceptible; S^−^ uninfected susceptible.

**Figure 6:**
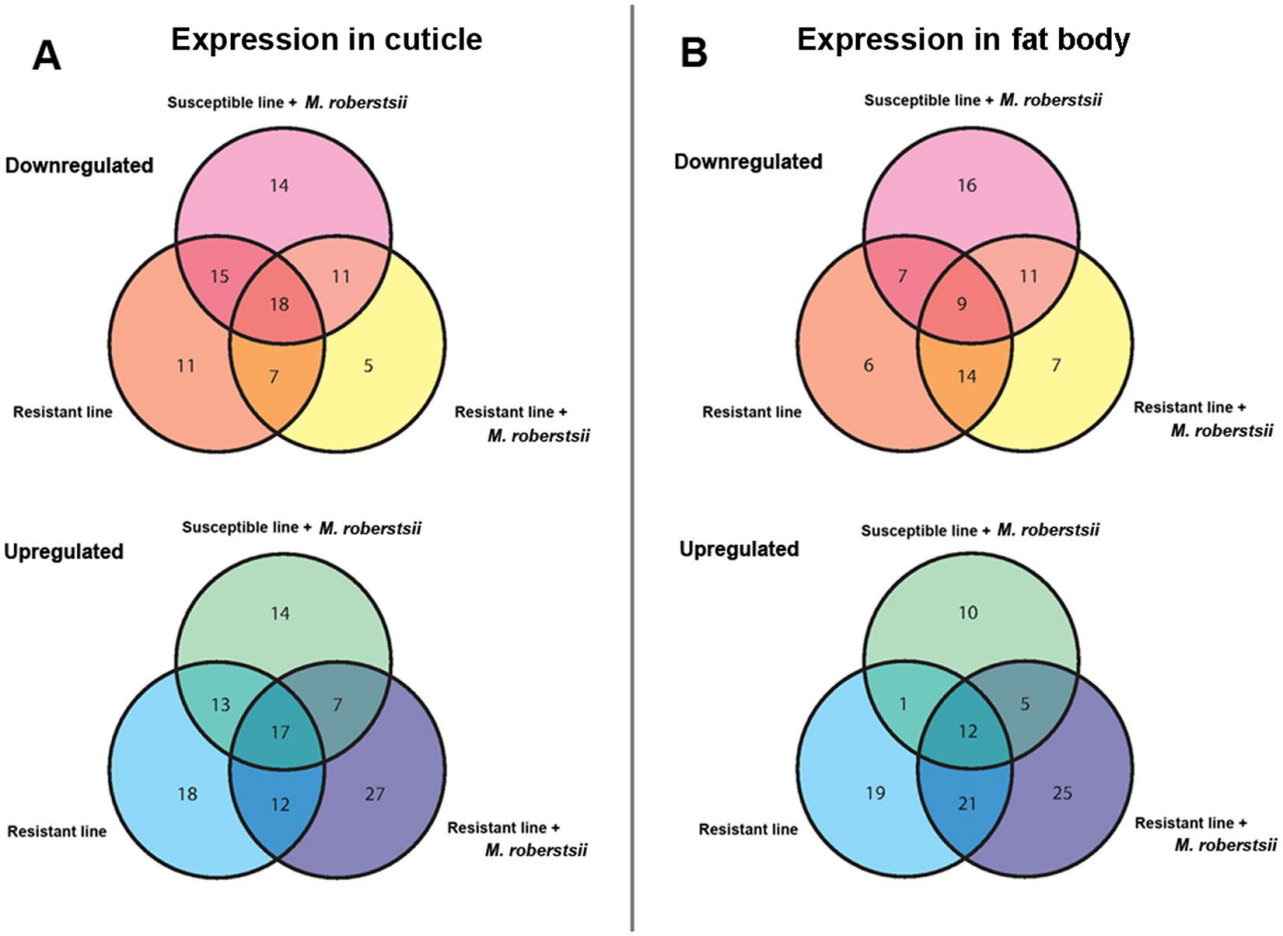
Venn diagram showing the differential expression of miRNAs in the cuticle and fat body of the four cohorts of *G*. *mellonella* larvae. The miRNA sequences were obtained from miRBase v21 and their expression profiles in (**A**) the cuticle and (**B**) the fat body were determined by microarray analysis. The fold differences of downregulated and upregulated miRNAs are shown relative to uninfected susceptible (S^−^) larvae (*p* < 0.01).

Duplicates expressed in both the R^+^ and S^+^ larvae were excluded. We then selected 173 miRNAs that were differentially expressed (*p* < 0.01) compared to S^−^ larvae in either the fat body (Fig. 4A–E) or the cuticle (Fig. 5A–E). We found 14, 27 and 18 miRNAs that were specifically upregulated, and 14, 5 and 11 miRNAs that were specifically downregulated, in the cuticle of S^+^, R^+^ and R^−^ larvae, respectively (Fig. 6A). Similarly, 10, 25 and 19 miRNAs were specifically upregulated and 16, 7 and 6 miRNAs were specifically downregulated in the fat body of the S^+^, R^+^ and R^−^ larvae, respectively (Fig. 6B). We identified a further 19 differentially expressed miRNAs in the cuticle (12 upregulated and 7 downregulated) by comparing R^+^ *vs* R^−^, and a further 16 differentially expressed miRNAs in the fat body (5 upregulated and 11 downregulated) by comparing R^+^ *vs* S^+^. Finally, we identified a further 35 differentially expressed miRNAs in the cuticle (17 upregulated and 18 downregulated) and a further 21 differentially expressed miRNAs in the fat body (12 upregulated and 9 downregulated) in all three test groups, i.e. [S^+^, R^+^ and R^−^] *vs* S^−^ (Fig. 6A–B).

### Identification of miRNA targets that confer resistance to *M*. *robertsii*

Selected miRNAs that were found to be differentially expressed between resistant and susceptible larvae were screened against the comprehensive *G*. *mellonella* transcriptome^28^. Targets were identified for 17 of these miRNAs and their biological processes and molecular functions were investigated *in silico*. Most of the target genes encoded proteinases and proteinase inhibitors, or were involved in cuticle formation, DNA damage, oxidoreductase activity, cell signaling, and metabolism (Fig. S2). RNAhybrid software was used to confirm selected targets by calculating the minimum free energy of miRNA–mRNA hybridization (Table S3). Complete seed sequence complementarity preceded miRNA–mRNA duplex formation, confirming the veracity of the targets.

## Discussion

Host–parasite coevolution is characterized by rapid mutual adaptations driven by the selection pressure imposed by each antagonist. Recent concepts in evolutionary biology predict that epigenetic mechanisms may mediate heritable shifts in phenotype within a few generations^7^. Here we imposed experimental selection pressure to enhance the resistance of *G*. *mellonella* against the parasitic fungus *M*. *robertsii*, allowing us to determine epigenetic differences between the resistant and susceptible lines of this model host. Our generation-spanning model confirmed that imposing selective pressure (parasite exposure) can experimentally shift a complex trait (resistance) in a heritable manner independent of changes in the DNA sequence^29,30^ because it is unlikely that mutation, recombination and population-wide spreading would occur during an experiment covering only a few generations. Given that parasite resistance is a complex trait involving numerous signaling pathways and effector genes, we anticipated changes in epigenetic mechanisms operating before and after transcription. Accordingly, we observed changes in DNA methylation and histone acetylation, as well as differentially expressed miRNAs in *G*. *mellonella* larvae with enhanced parasite resistance.

DNA methylation is found in many insects, with diverse profiles across insect taxa^31^. This epigenetic mechanism can affect gene expression in insects positively or negatively, and generally targets constitutively expressed genes, whereas tissue-specific genes tend to be sparsely methylated^32–34^. We observed small but distinct differences in DNA methylation between R^−^ and S^−^ larvae, and between the cuticle and fat body in the S^−^ line. Such differences correlated with changes in the expression of tissue-specific genes that control innate immunity in *G*. *mellonella*, facilitating the transition from a susceptible to a resistant phenotype within a few generations in response to repeated encounters with *M*. *robertsii*. The evolution of resistance against a particular pathogen often includes a more vigilant systemic immune response compared to a susceptible population. Compared to S^−^ larvae, DNA methylation levels were rather low in the fat body of R^−^ larvae. The fat body expresses antimicrobial peptides (AMPs), and the basal expression of AMPs was elevated in larvae resistant to the fungus *Beauveria bassiana* compared to susceptible larvae^35^. Here we suggest that DNA methylation could negatively affect AMP gene expression in the fat body of resistant larvae as previously observed in *Bombyx mori* following viral infection^34^. However, the increase in DNA methylation in the cuticle of R^−^ larvae compared to S^−^ larvae suggests that this epigenetic mechanism does not suppress AMPs induced specifically in the cuticle to prevent penetration by *M*. *robertsii*^35^. The expression of innate immunity-related genes in the cuticle and fat body of resistant larvae remained at basal levels following infection with *B*. *bassiana*, whereas the same genes were strongly upregulated in infected susceptible larvae^35^. Here we found that the genes encoding DNA methyltransferase 1-associated protein 1 and DNA cytosine-5 methyltransferase were differentially expressed among the four cohorts we tested. DNA methyltransferase 1-associated protein is a co-repressor that stimulates DNA methylation globally and locally at double-strand break repair sites^36^. The downregulation of DNA cytosine-5 methyltransferase in R^+^ larvae relative to R^−^ larvae, and the upregulation of the same gene in S^+^ larvae relative to S^−^ larvae, indicates transcriptional reprogramming associated with the evolution of resistance^36^. These results are supported by our earlier generation-spanning experiments, in which *G*. *mellonella* was selected for resistance against *Bacillus thuringiensis*. The resistant line showed similar shifts in DNA methylation when compared with the susceptible control population^7^.

In insects, DNA methylation interacts with other epigenetic mechanisms such as histone acetylation to control gene expression, e.g. to regulate behavioral plasticity and social behavior in Hymenoptera^37,38^. We propose that DNA methylation, either alone or jointly with other epigenetic mechanisms, influences the ability of *G*. *mellonella* to evolve resistance to *M*. *robertsii*. In agreement with our hypothesis, we show that the evolution of resistance to *M*. *robertsii* in *G*. *mellonella* is associated with a tissue-specific increase in the acetylation of histone H3. Gene expression is regulated by the addition or removal of acetyl groups on histones, a mechanism that is tightly controlled by the opposing activities of HDACs and HATs. We found that HDACs and HATs were upregulated in the R^−^ larvae but were downregulated in R^+^ larvae. The dysregulation of HDACs and HATs favors microbial pathogenesis^39,40^, and can promote the evolution of resistance to ionizing radiation in lepidopteran Sf9 cell lines^41^. Acetylation of the core histone H3 is involved in the regulation of chromatin structure and the recruitment of transcription factors to promoters. A tissue-specific increase in H3 acetylation in *G*. *mellonella* was associated with the evolution of resistance to the bacterial pathogen *B*. *thuringiensis*, indicating that this epigenetic mechanism regulates the transcriptional activation of immunity-related genes^35,42^.

In eukaryotes, miRNAs can regulate gene expression post-transcriptionally, and are conserved among different insect orders^43,44^. To investigate the role of *G*. *mellonella* miRNAs in the evolution of resistance against *M*. *robertsii*, we used microarrays imprinted with probes representing more than 2000 insect miRNA sequences deposited in miRBase. Using this approach as previously described^26^, we analyzed the expression profile of 2621 unique mature miRNAs and identified candidates that were modulated in the cuticle and fat body in each of the larval cohorts. For example, in R^+^ larvae we detected the expression of miR-6498-5p, miR-5729b-3p, miR-3885-5p, miR-8516 and miR-9377-3p specifically in the cuticle, and miR-277-3p, miR-13-3p, miR-2789, miR-3826-5p and miR-310a specifically in the fat body. We also detected miRNAs such as miR-278-3p and miR-2767, which are expressed universally in *G*. *mellonella* during selection for resistance to bacterial (*B*. *thuringiensis*) or fungal (*M*. *robertsii*) pathogens^7^. Furthermore, we detected the expression of miR-9b-3p, miR-87a-3p and miR-184 in susceptible larvae, and miR-9377-3p, miR-10-5p, miR-9894, miR-6492, miR-3756 and miR-4968-3p in resistant larvae, infected with *M*. *robertsii* (this study) or *B*. *thuringiensis*. Other miRNAs, such as miR-3789, were expressed in both resistant and susceptible *G*. *mellonella* larvae. Similarly, miR-6006-5p was expressed in both R^+^ and R^−^ larvae. These miRNAs appear to have roles that contribute to the adaptation of insects against pathogens during selection for resistance.

The target mRNAs for the miRNAs were identified *in silico* using the *G*. *mellonella* transcriptome, given the absence of a complete annotated genome sequence^28^. Putative 3′-UTRs were aligned with the modulated miRNAs, revealing 17 miRNAs and 44 corresponding mRNA targets. Functions were tentatively assigned based on annotated sequences in other insects. The large number of miRNAs that are differentially expressed between susceptible and resistant *G*. *mellonella* suggests that resistance involves the post-transcriptional regulation of gene expression, although more work is required to pinpoint the functions of specific miRNAs.

Recently, epigenetic mechanisms were shown to control the expression of genes encoding virulence-associated proteinases in *M*. *robertsii* which are induced by the presence of host-derived antifungal peptides or proteinase inhibitors^18^. Strikingly, host–parasite coevolution resulted in the ability of both partners in this antagonistic system to sense the presence of molecules produced by the other, resulting in a complex series of attacks and counterattacks that indicate communication between parasitic fungi and their insect hosts^18^.

## Conclusions

Our study supports the postulated role of epigenetics in the rapid manifestation of heritable traits in response to environmental stimuli^7^. The evolution of resistance against fungal pathogens involves complex transcriptional reprogramming in the insect host, which is reflected by the determined contribution of different epigenetic mechanisms operating before and after transcription. These epigenetic mechanisms mediate the translation of selection pressure into a heritable phenotype (enhanced resistance). The emerging insights into the complex molecular interactions occurring at the genetic and the epigenetic levels during host–parasite coevolution also support the hypothesis that antagonistic coevolution accelerates molecular evolution. Our work will encourage scientists to investigate the inheritance of epigenetic changes during evolution thereby addressing debatable issues such as whether epigenetic mechanisms are a cause or effect of the evolution of complex traits.

## Methods

### Insect rearing, *M*. *robertsii* infections and design of experiments

*G*. *mellonella* larvae were reared in isolation (28 °C, 60% relative humidity, 12-h photoperiod) and were fed on artificial medium (AM) comprising 22.5% corn meal, 12.5% honey, 12.5% glycerol, 12.5% beeswax, 10% wheat flour, 12.5% milk solids, 5% yeast and 12.5% water. The entomopathogenic fungus *M*. *robertsii* (strain MB-1) from the ISEA SB RAS collection was cultured as described in the Electronic Supplementary Information. Artificial selection was carried out as previously described^6^ (see Electronic Supplementary Information for the detailed protocol) resulting in resistant and susceptible lines. Fifth-instar larvae from the two lines were compared in generation six to determine their susceptibility to *M*. *robertsii* and to investigate the associated epigenetic changes. Larvae from both lines were inoculated with *M*. *robertsii* conidia, resulting in the infected resistant (R^+^) and infected susceptible (S^+^) cohorts, which were compared to uninfected resistant (R^−^) and uninfected susceptible (S^−^) controls (see Electronic Supplementary Information for the protocol). The LC_50_ of line R^+^ was divided by the LC_50_ of line S^+^ to calculate the degree of resistance. In a parallel study, fifth-instar larvae from all four cohorts were collected 48 h post-exposure and the cuticle and fat body were dissected for the isolation of DNA, RNA and histones (n = 9 larvae per treatment per line). All experiments were carried out in triplicate. The fat body and cuticle were dissected from at least three chilled, surface-sterilized larvae per treatment and crushed with a pestle in cell lysis solution. Resistant and susceptible larvae were sampled 48 h after topical application of *M*. *robertsii* conidia and uninfected control larvae were sampled in parallel.

### Measurement of global changes in DNA methylation

DNA was isolated using the DNA sorb B kit (AmpliSens, Russia). The relative percentage of methylated DNA was estimated using the MethylFlash Methylated DNA Quantification kit (EpigenTek, USA). DNA isolated from the cuticle and fat body was coated onto 96-well plates (100 ng per sample). The methylated DNA was detected using capture and detection antibodies by measuring the absorbance at 450 nm in a microplate reader (BioTek, USA).

### Measurement of global histone H3 acetylation

Global histone H3 acetylation levels in resistant and susceptible larvae were determined using the EpiQuik™ global histone H3 acetylation assay kit (EpigenTek, USA). Samples were extracted in three volumes of buffer with glycerol on ice for 5 min, and the supernatant was mixed with 100% trichloroacetic acid and incubated on ice for 30 min before centrifugation (10 min, 13,523 x g, 4 °C). The pellet was washed twice with acetone and dissolved in water. The histone protein concentration was estimated using the bicinchoninic acid (BCA) method and the extract was divided into aliquots for storage at −80 °C prior to analysis. Following extraction, the histone proteins (1–2 μg) were coated onto the strip wells and acetylated histone H3 was detected using a high-affinity antibody. The ratio in resistant and susceptible larvae was estimated using a horseradish peroxidase (HRP)-conjugated secondary antibody and the colorimetric signal was quantified by measuring the absorbance at 450 nm.

### RT-PCR analysis

Total RNA was prepared by collecting samples in RNA-later (Ambion, UK), homogenizing under liquid nitrogen and extracting the RNA using TRIzol Reagent (Invitrogen, USA). RNA concentrations were determined by spectrophotometry. Relative mRNA expression levels were determined by RT-PCR as previously described, using RNA from the cuticle and fat body of each larval cohort^22^. Specific target mRNAs and genes coding for HDACs, HATs and DNMTs by RT-PCR were amplified as previously described^28^ using the following primer sequences: histone deacetylase 8-fwd/rev (5′-GAT ACA GTG TGG TGC GGA TG-3′/5′-GCA ACA AGA GCA GTG ATG GA-3′), histone deacetylase 8 isoform 2-fwd/rev (5′-TCT TCA TCT TGT GGG GTT GA-3′/5′-GCG GGC TTC TTT AAT ACA CG-3′), histone deacetylase complex subunit-fwd/rev (5′-ACT TCA GGC GAG TCC ATC AG-3′/5′-ACA ACG AAC GTT GCA GAC AG-3′), histone deacetylase complex subunit sap18-fwd/rev (5′-GAA ACT CGA CGC AAA GGA AC-3′/5′-CTC ATT GGT GGA GGC ATT CT-3′), histone acetyltransferase 1-fwd/rev (5′-CGC ATT GTG CCA TTT AGT TG-3′/5′-TGA AGG CTT CCT GCA CTG TA-3′), histone acetyltransferase tip60- fwd/rev (5′-CGC GAA ATG GTA ACA AAC AG-3′/5′-TGG AGA GCC ACA TAA CAA CTG-3′), histone acetyltransferase type b catalytic-fwd/rev (5′-CCT GAA CGT TGT GGA CAT CA-3′/5′-CGC GCC TGT TTC TTG TTT AT-3′), DNA cytosine-5 methyltransferase-fwd/rev (5′-GTG GTA TGC ACT GTG GAT GG-3′/ 5′-AAG GCT GAC ATG GTG GAG AC-3′), DNA methyltransferase 1-associated protein 1-fwd/rev (5′-CAA ACA AAG GCG AAG CTA GG-3′/5′- CCA TCA AAT GAT CGG TTT CC-3′) and the housekeeping gene 18 *S* rRNA-fwd/rev (5′-ATG GTT GCA AAG CTG AAA CT-3′/5′-TCC CGT GTT GAG TCA AAT TA-3′).

### Microarray analysis of miRNAs

Microarray analysis of miRNAs, including the provision of reagents, experimental procedures and data analysis, was carried out by LC Sciences, Houston, TX, USA. Total RNA (2 µg) was extended using poly(A) polymerase and ligated to an oligonucleotide tag for fluorescent dye staining. Hybridization was performed overnight on a µParaflo microfluidic chip using a micro-circulation pump (Atactic Technologies)^45^ in 100 µL 6 x SSPE buffer (0.90 M NaCl, 60 mM Na_2_HPO_4_, 6 mM EDTA, pH 6.8) containing 25% formamide at 34 °C. After hybridization, tag-conjugating Alexa Fluor647 dye was circulated through the microfluidic chip. Fluorescence images were captured using a GenePix 4000B laser scanner (Molecular Devices) and digitized using Array-Pro image analysis software (Media Cybernetics). After background subtraction and normalization using a locally-weighted regression filter^26,46,47^, p values were corrected for multiple testing using the false discovery rate calculated using the Benjamini-Hochberg procedure^48^.

### Prediction of miRNA targets

The miRNA targets were predicted by finding open reading frames (ORFs) in all contigs in the sequenced *G*. *mellonella* transcriptome using the ‘Find next ORF’ option in the sequence alignment editor BioEdit v7.2.5^26^. Nucleotide sequences at the 3′ end of individual contigs lying outside confirmed ORFs were considered as potential 3′ UTRs. The miRNA sequences (5′ → 3′ direction) were first converted to DNA sequences and then reverse complemented, with 2–8 nucleotides (5′ → 3′ direction) of the reverse complementary sequence considered as the seed region (Fig. S3). Seed sequences matching identified 3′ UTRs of *G*. *mellonella* transcriptome sequences were considered targets for complementary miRNA sequences. The Gene Ontology categories of the identified contigs were listed by consulting the UniProt database and a previous report^28^ (Table S4). The biological processes targeted by miRNAs in the resistant and susceptible lines were summarized using Cytoscape v3.2.1 (Fig. S2). The structure of miRNA–mRNA duplexes was visualized using the RNAhybrid tool provided by the Bielefeld Bioinformatics Server^49^ (Table S3).

### Data analysis

Data were analyzed using GraphPad Prism v4.0 (GraphPad Software Inc., USA) and Microsoft Excel 2013 (Microsoft Corp., USA). All experiments except microarray analysis were performed a minimum of three times. Data were checked for normal (Gaussian) distribution using the Agostino-Pearson omnibus test. Cox’s proportional hazards survival regression was used to quantify differences in mortality rates after fungal infection between selected and unselected larvae. Significant differences between pairs of values representing DNA methylation and histone acetylation levels were compared using a paired Student’s *t* test. Similarly, pairwise comparisons of miRNA expression levels were carried out using a paired Student’s *t* test and analysis of variance (ANOVA). Differences in miRNA expression levels were considered significant at *p* < 0.01 and in all other experiments the significance threshold was set to *p* < 0.05.

## Electronic supplementary material

Supplementary Information

Table S1

Table S2

## Data Availability

All data are accessible in the Supplementary Information.
