# Supplementary material for: Epigenetic mechanisms mediate the experimental evolution of resistance against parasitic fungi in the greater wax moth Galleria mellonella
Source: Sci Rep. 2019 Feb 7;9:1626. doi: 10.1038/s41598-018-36829-8 (PMC6367475; doi:10.1038/s41598-018-36829-8)
Supplement: Supplementary file 1 — Supplementary Information [file 41598_2018_36829_MOESM1_ESM.pdf]

Epigenetic mechanisms mediate the experimental evolution of resistance against parasitic fungi in the greater wax moth *Galleria mellonella*

Krishnendu Mukherjee[1#], Ivan Dubovskiy[2#], Ekaterina Grizanova[2], Rüdiger Lehmann[1], Andreas Vilcinskas[1,3*]

[1]Fraunhofer Institute for Molecular Biology and Applied Ecology, Department of Bioresources, Winchester Str. 2, 35394 Giessen, Germany

[2]Novosibirsk State Agrarian University, Dobrolubova 160, 630039 Novosibirsk, Russia

[3]Institute for Insect Biotechnology, Justus-Liebig University of Giessen,

Heinrich-Buff-Ring 26-32, 35392 Giessen, Germany

Tel: +49 641 99 37600

Fax: +49 641 99 37609

*** Correspondence to andrea.vilcinskas@agrar.uni-giessen.de**

#These authors contributed equally to this work.

Supplementary Figures, Methods

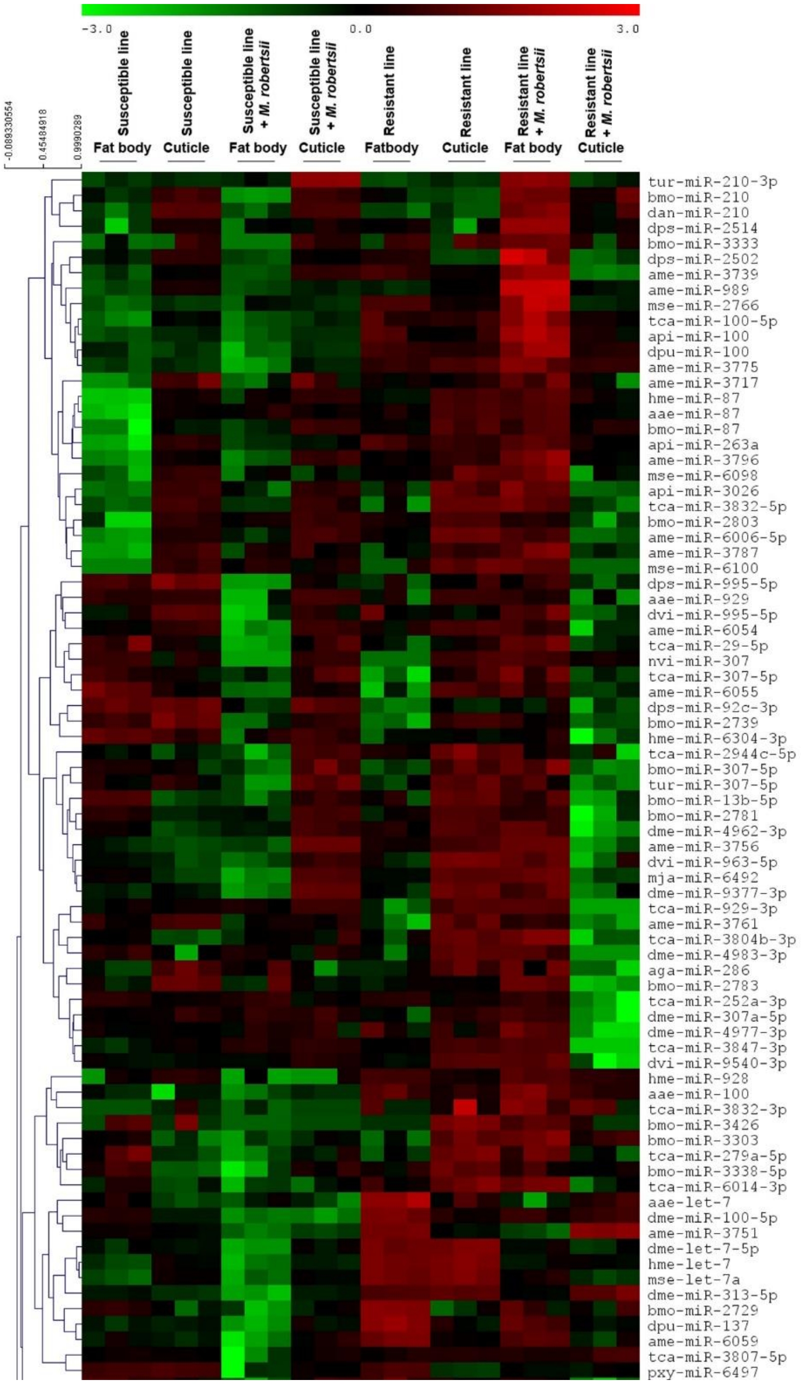

Supplementary Fig. S1. **Comparative expression profiling of selected miRNAs in infected/uninfected resistant and susceptible *G. mellonella* larvae.** The microarray heat map was generated following microarray hybridization, statistical analysis and hierarchical clustering. The heat map highlights a set of differentially-expressed miRNAs between fat body and cuticle of uninfected and infected susceptible and resistant lines with red showing upregulated genes and green showing downregulated genes. The log score of each fold change is indicated.

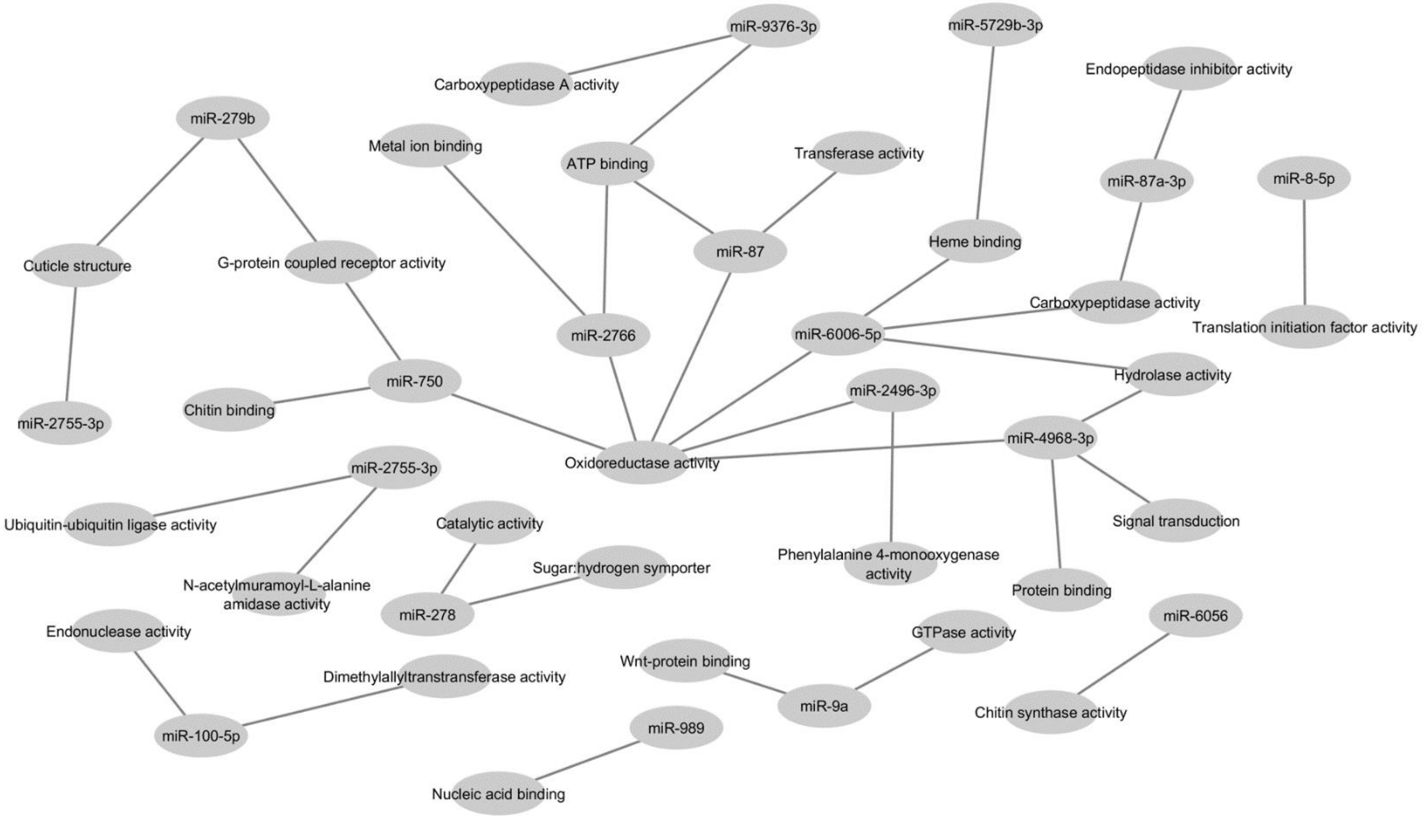

Supplementary Fig. S2. **The molecular functions targeted by miRNAs in *G. mellonella*.** The network diagram generated with Cytoscape shows molecular functions in infected/uninfected resistant and susceptible larvae that are targeted by differentially-expressed miRNA sequences obtained from miRBase. Connecting lines (edges) are used to indicate miRNA targets identified in this study by consulting Gene Ontology terms related to *G. mellonella* transcriptome sequences[29] and the UniProt database.

Supplementary Table S3: miRNA target prediction by RNAhybrid

	**Predicted mRNA-miRNA helix**
Target: contig16200_1.r1.exp<br>length: 1856<br>miRNA : dme-miR-4968-3p<br>length: 22<br>mfe: -27.1 kcal/mol<br>p-value: 1.000000e+00<br>position 1497	target 5' A U 3'<br>UGUUGUUGCUGCU<br>ACGACGACGACGA<br>miRNA 3' AG CAACGAC 5'
Target: contig21563_1.exp<br>length: 2106<br>miRNA : dme-miR-4968-3p<br>length: 22<br>mfe: -26.6 kcal/mol<br>p-value: 1.000000e+00<br>position 1901	target 5' A A A CUUUUUUUCAACCA AC A 3'<br>UC GC GCU GU GCUGUUGCUG<br>AG CG CGA CG CGACAACGAC<br>miRNA 3' A A A 5'
Target: GME-string_Contig_6635.0<br>length: 1148<br>miRNA : dme-miR-4968-3p<br>length: 22<br>mfe: -23.3 kcal/mol<br>p-value: 1.000000e+00<br>position 989	target 5' U ACACUUUACAAUUU GUAAUUG A 3'<br>UUUGUUGCUGU UGU UGC<br>AGACGACGACG ACG ACG<br>miRNA 3' ACA AC 5'
Target: contig17067_1.exp<br>length: 1402<br>miRNA : dme-miR-4968-3p<br>length: 22<br>mfe: -17.6 kcal/mol<br>p-value: 1.000000e+00<br>position 1377	target 5' A A 3'<br>UGUUGCUG<br>ACGACGAC<br>miRNA 3' AGACGACG AACGAC 5'
Target: contig01373_1.f1.exp<br>length: 1537<br>miRNA : isc-miR-278<br>length: 22<br>mfe: -23.7 kcal/mol<br>p-value: 1.000000e+00<br>position 1295	target 5' U C U AG A 3'<br>GAAUGGA CGA GAU CACC<br>UUUGCCU GCU UUA GUGG<br>miRNA 3' U GG CU 5'
Target: contig21748_1.f1.exp<br>length: 2636<br>miRNA : isc-miR-278<br>length: 22<br>mfe: -12.2 kcal/mol<br>p-value: 1.000000e+00<br>position 2410	target 5' A UC UG U 3'<br>ACG AUUCU U<br>UGC UAGGG G<br>miRNA 3' UUUGCC UUU UG CU 5'
Target: GME-string_Contig_1981.0<br>length: 3279<br>miRNA : mse-miR-2766<br>length: 22<br>mfe: -4.5 kcal/mol	target 5' G A 3'<br>UAU<br>GUG<br>miRNA 3' UGG GUAAGCUGUUCUGACU 5'

p-value: 1.000000e+00<br>position 3109	
Target:<br>contig20381_1.f1.exp<br>length: 2170<br>miRNA : mse-miR-2766<br>length: 22<br>mfe: -22.3 kcal/mol<br>p-value: 1.000000e+00<br>position 1941	target 5' G CAU UAU A 3'<br>UCAUUA UUUG UAAGACUGA<br>GGUGGU AAGC GUUCUGACU<br>miRNA 3' UG U 5'
Target: contig15219_1.exp<br>length: 1022<br>miRNA : mse-miR-2766<br>length: 22<br>mfe: -17.2 kcal/mol<br>p-value: 1.000000e+00<br>position 875	target 5' G G AA G UCA A 3'<br>AUU A AU UG AAGACUGA<br>UGG U UA GC UUCUGACU<br>miRNA 3' G GG A UG 5'
Target:<br>contig16750_1.f1.exp<br>length: 2464<br>miRNA : mse-miR-2766<br>length: 22<br>mfe: -20.0 kcal/mol<br>p-value: 1.000000e+00<br>position 2366	target 5' G G GG U GCCAA U 3'<br>U CACU UG ACA AGACUGA<br>G GUGG GC UGU UCUGACU<br>miRNA 3' U G UAA 5'
Target:<br>contig19310_1.f1.exp<br>length: 3464<br>miRNA : dme-miR-100-5p<br>length: 22<br>mfe: -21.3 kcal/mol<br>p-value: 1.000000e+00<br>position 3042	target 5' G U CCAA G 3'<br>AUGA UUC GUUUACGGGUU<br>UGUU AAG UAAAUGCCCAA<br>miRNA 3' G C CC 5'
Target:<br>contig20454_1.f1.exp<br>length: 2044<br>miRNA : dme-miR-100-5p<br>length: 22<br>mfe: -16.8 kcal/mol<br>p-value: 1.000000e+00<br>position 1723	target 5' A A 3'<br>UACGGGU<br>AUGCCCA<br>miRNA 3' GUGUUCAAGCCUAA A 5'
Target: contig02833_1.exp<br>length: 1232<br>miRNA : bmo-miR-87<br>length: 21<br>mfe: -18.3 kcal/mol<br>p-value: 1.000000e+00<br>position 765	target 5' G CU A 3'<br>UGAAG UUUGCUCA<br>ACUUU AAACGAGU<br>miRNA 3' UGUGUGG C 5'
Target: contig02824_1.exp<br>length: 686<br>miRNA : bmo-miR-87<br>length: 21<br>mfe: -15.2 kcal/mol<br>p-value: 1.000000e+00<br>position 593	target 5' A UUA UU U 3'<br>GC ACUU UUUGCUC<br>UG UGGA AAACGAG<br>miRNA 3' UG CUUUC U 5'

Target: GME-string_Contig_374.0<br>length: 1081<br>miRNA : bmo-miR-87<br>length: 21<br>mfe: -17.8 kcal/mol<br>p-value: 1.000000e+00<br>position 951	target 5' C UG UAUAACUACU A 3'<br>U UUGA UUUGCUCA<br>G GACU AAACGAGU<br>miRNA 3' UGU UG UUC 5'
Target: GME-string_Contig_1571.0<br>length: 1437<br>miRNA : pxy-miR-9a<br>length: 21<br>mfe: -14.1 kcal/mol<br>p-value: 1.000000e+00<br>position 1100	target 5' C AAA U 3'<br>AC UAGCUUUG<br>UG AUCGAAAU<br>miRNA 3' UUGAGGCCAU G 5'
Target: GME-string_Contig_3530.0<br>length: 2186<br>miRNA : pxy-miR-9a<br>length: 21<br>mfe: -15.2 kcal/mol<br>p-value: 1.000000e+00<br>position 1564	target 5' A G U 3'<br>UAAUC UAGCUUU<br>AUUGG AUCGAAA<br>miRNA 3' UUGAGGCC U 5'
Target: contig19490_1.f1.exp<br>length: 2513<br>miRNA : pxy-miR-9a<br>length: 21<br>mfe: -17.0 kcal/mol<br>p-value: 1.000000e+00<br>position 1927	target 5' U AA UUU A 3'<br>AUUC GUAGC CUAGU<br>UGAG CAUUG GAUCG<br>miRNA 3' U GC AAAU 5'
Target: GME-string_Contig_1493.0<br>length: 2344<br>miRNA : ame-miR-6006-5p<br>length: 19<br>mfe: -23.3 kcal/mol<br>p-value: 1.000000e+00<br>position 2002	target 5' A GA C 3'<br>UCUU UCCCAGUUAU<br>AGAG AGGGUCAAUA<br>miRNA 3' GG AGA 5'
Target: contig21833_1.exp<br>length: 1869<br>miRNA : ame-miR-6006-5p<br>length: 19<br>mfe: -14.9 kcal/mol<br>p-value: 1.000000e+00<br>position 1792	target 5' A ACAAGAA C 3'<br>CU CCAGUUA<br>GA GGUCAAU<br>miRNA 3' G GAGAGAAG A 5'
Target: contig16741_1.f1.exp<br>length: 1825<br>miRNA : ame-miR-6006-5p<br>length: 19<br>mfe: -17.0 kcal/mol<br>p-value: 1.000000e+00<br>position 1042	target 5' A U U 3'<br>UUC CCAGUUAU<br>AAG GGUCAAUA<br>miRNA 3' GGAGAGAG 5'

Target: GME-<br>string_Contig_6193.0<br>length: 1632<br>miRNA : ame-miR-6006-5p<br>length: 19<br>mfe: -20.6 kcal/mol<br>p-value: 1.000000e+00<br>position 848	target 5' A GCAUAUGAU A A 3'<br>CUUUC CU UC CCAGUUAU<br>GAGAG GA AG GGUCAAUA<br>miRNA 3' G A 5'
Target:<br>contig00467_1.f1.exp<br>length: 2048<br>miRNA : tca-miR-87a-3p<br>length: 21<br>mfe: -22.3 kcal/mol<br>p-value: 1.000000e+00<br>position 1377	target 5' U G G UAUAG C 3'<br>CACA U GAG UUGCUCAC<br>GUGU G CUU AACGAGUG<br>miRNA 3' G A UGA 5'
Target:<br>contig02728_1.f1.exp<br>length: 1846<br>miRNA : tca-miR-87a-3p<br>length: 21<br>mfe: -20.2 kcal/mol<br>p-value: 1.000000e+00<br>position 1783	target 5' G A 3'<br>UAUUUGAG UUGCUCA<br>GUGGACUU AACGAGU<br>miRNA 3' GU UGA G 5'

Insect rearing and selection

The experimental and stock populations of *G. mellonella* were reared in strict isolation at 28°C, 60% relative humidity, with a 12:12h light:dark cycle, and fed on artificial medium (AM) containing 22.5% corn meal, 12.5% honey, 12.5% glycerol, 12.5% beeswax, 10% wheat flour, 12.5% milk solids, 5% yeast and 12.5% water. At the start of the artificial selection procedure, the laboratory insect population was split into two separate lines. Imagoes (100 males; 100 females) were placed in a 10L plastic tank at 28 °C in the dark to lay eggs on AM. One line was then subjected to selection (Selected line, S) and the second was left as the non-selected line (NS). At least 1000 5th instar larvae were used for selection for each of the first three generations followed by at least 400 larvae for subsequent selections. In each selection cycle, 400 S-line larvae were infected by 1×10^7*M. robertsii* conidia to establish a mycosis (LC_{40-50}). The NS line insects were always kept under fungus-free conditions. To initiate the S-line next generation, 200 of the surviving larvae were collected and allowed to breed. This number of insects per population is enough to reduce the risks of inbreeding and genetic drift[1]. Two hundred uninfected NS-line insects (of exactly the same age as those used for the S-line) were also collected and allowed to breed. The experiment was continued for 6 generations.

Fungus cultivation

M. robertsii (strain MB-1) was isolated from soil in Novosibirsk region (West Siberia) at 2009 year[2]. Fungi were grown on Sabouraud's dextrose agar (SDA) at 25°C for 14 d. Conidia were harvested by scraping from sporulating cultures, air-dried at RT for 1 week and stored at 4°C. For topical infections, conidia were suspended in sterile 0.03% Tween–80 and vortexed for 1 min. Viability of conidia was verified by incubation of these propagules on SDA and determining the percentage germination. Only suspensions with at least 99% germination were used.

Fungal infections

Each insect was dipped in an aqueous suspension of the *M. robertsii* for 10 s using a concentration of 1×10^7 conidia / ml. Dipped insects were kept in Petri dishes (10 larvae / dish) until sacrificed. The uninfected control insects were dipped into distilled water (n=60). Larvae were observed daily for (up to pupation) 10 days. All dead animals were removed and examined to confirm the cause of death. All animals used in these experiments were 5th instar larvae raised in the same cohort. The experiment was repeated independently three times. The total number of individuals used from each line was 300.

References

1. Roy, B. A. & Kirchner, J. W. Evolutionary dynamics of pathogen resistance and tolerance. *Evolution* **54,** 51-63 (2000).

2. Kryukov, V. et al. Ecological preferences of *Metarhizium* spp. from Russia and neighboring territories and their activity against Colorado potato beetle larvae. *Journal of Invertebrate Pathology* **149,** 1-7 (2017). http://dx.doi.org/10.1016/j.jip.2017.07.001

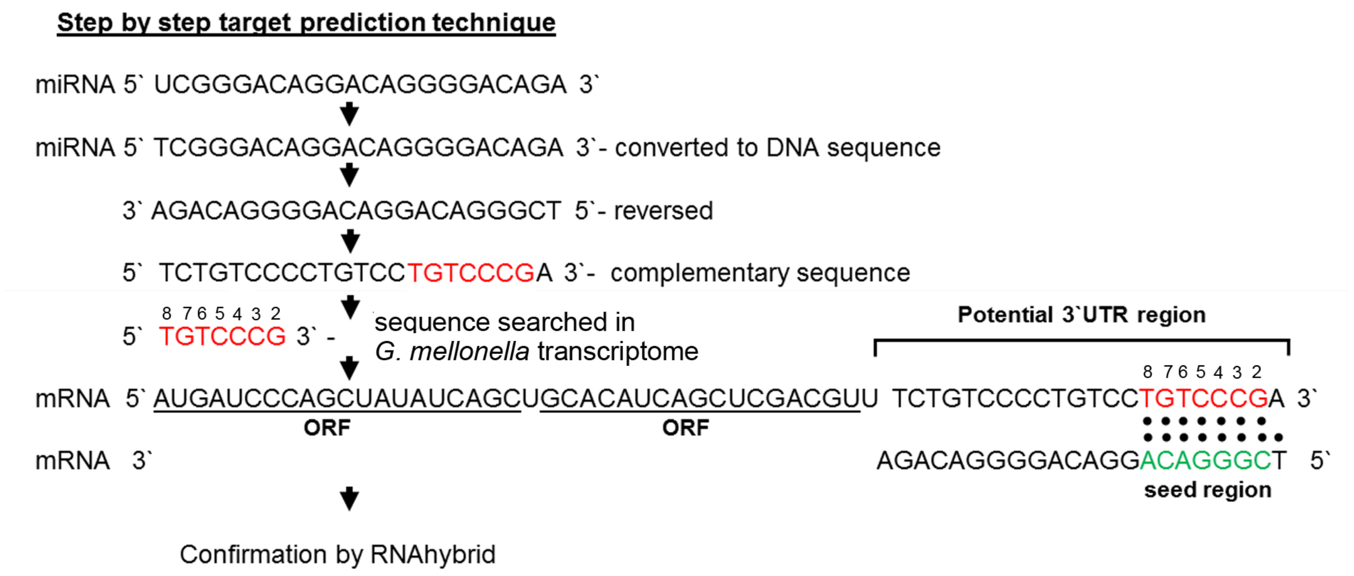

Supplementary Fig. S3. **Schematic illustration of the strategy for predicting miRNA targets using *G. mellonella* transcriptome.**

Supplementary table S4: GO terms of the identified mRNA targets of miRNAs

**MicroRNA**	**Target**	**Molecular function**	**Biological process**
dme-miR-4968-3p	Contig21563_1.exp	Calcium channel activity	ER calcium ion homeostasis
dme-miR-4968-3p	Contig16200_1.r1.exp	Protein binding	
dme-miR-4968-3p	GME-string_Contig_6635.0	Thiol oxidase activity	
dme-miR-4968-3p	Contig17067_1.exp	Hydrolase activity	Lipid catabolic process
dme-miR-9376-3p	Contig15458_1.f1.exp	ATP binding; protein serine/threonine kinase activity	Protein amino acid phosphorylation

dme-miR-9376-3p	GME-string_Contig_1591.0	Carboxypeptidase A activity	Proteolysis
isc-miR-278	Contig01373_1.f1.exp	Sugar:hydrogen symporter activity	Carbohydrate transport
isc-miR-278	Contig21748_1.f1.exp	Catalytic activity	Metabolic process
mse-miR-2766	GME-string_Contig_1981.0	Oxidoreductase activity	
mse-miR-2766	Contig20381_1.f1.exp	Metal ion binding	
mse-miR-2766	Contig15219_1.exp		Response to stress
mse-miR-2766	Contig16750_1.f1.exp	ATP binding	Protein folding
dme-miR-100-5p	Contig19310_1.f1.exp	Endonuclease activity	Nucleotide-excision repair
dme-miR-100-5p	Contig20454_1.f1.exp	Dimethylallyltranstransferase activity	Steroid biosynthetic process

dme-miR-100-5p	Contig15219_1.exp		Response to stress
bmo-miR-87	Contig02833_1.exp	ATP binding	Protein folding
bmo-miR-87	Contig19596_1.exp	Transferase activity, transferring hexosyl groups	Metabolic process
bmo-miR-87	Contig02824_1.exp	Protein disulfide oxidoreductase activity	Glycerol ether metabolic process
bmo-miR-87	GME-string_Contig_374.0		Juvenile hormone binding
pxy-miR-9a	GME-string_Contig_3530.0		Proteolysis
pxy-miR-9a	Contig19490_1.f1.exp	Wnt-protein binding	Wnt signaling pathway
pxy-miR-9a	GME-string_Contig_1571.0	GTPase activity	Protein transport
ame-miR-6006-5p	GME-string_Contig_1493.0		Transport

ame-miR-6006-5p	Contig21424_1.exp	Hydrolase activity	Metabolic process
ame-miR-6006-5p	Contig21833_1.exp	Heme binding	Electron transport
ame-miR-6006-5p	Contig16741_1.f1.exp	Oxidoreductase activity	Fatty acid biosynthetic process
ame-miR-6006-5p	GME-string_Contig_6193.0	Serine-type carboxypeptidase activity	Proteolysis
tca-miR-87a-3p	Contig00467_1.f1.exp	Serine-type carboxypeptidase activity	Proteolysis
tca-miR-87a-3p	Contig02728_1.f1.exp	Serine-type endopeptidase inhibitor activity	
ame-miR-750	Contig01077_1.exp	Oxidoreductase activity	

ame-miR-750	Contig15746_1.exp	G-protein coupled receptor activity	Cell surface receptor signaling pathway
ame-miR-750	Contig02423_1.exp	Chitin binding	Carbohydrate metabolic process
mse-miR-989	GME-string_Contig_4453.0	Nucleic acid binding	
mse-miR-989	Contig03221_1.exp		Proteolysis
dme-miR-2496-3p	Contig00345_1.exp	Phenylalanine 4-monooxygenase activity	L-phenylalanine catabolic process
dme-miR-2496-3p	Contig14976_1.exp	Oxidoreductase activity	
ame-miR-6056	Contig05033_1.exp	Chitin synthase activity	Chitin biosynthetic process
tur-miR-5729b-3p	Contig18067_1.r1.exp	Heme binding	

api-miR-279b	Contig02910_2.r1.exp	G-protein coupled receptor activity	Cell surface receptor signaling pathway
api-miR-279b	GME-string_Contig_1151.0	Structural constituent of cuticle	
api-miR-279b	Contig20024_1.f1.exp		Cell redox homeostasis
bmo-miR-2755-3p	GME-string_Contig_1310.0	Structural constituent of cuticle	
bmo-miR-2755-3p	GME-string_Contig_3363.0|	Ubiquitin-ubiquitin ligase activity	Ubiquitin-dependent protein catabolic process
bmo-miR-2755-3p	Contig02387_1.f1.exp	N-acetylmuramoyl-L-alanine amidase activity	Peptidoglycan catabolic process
dme-miR-8-5p	GME-string_Contig_1938.0	Translation initiation factor activity	Transcription initiation from RNA polymerase II promoter
